# Mapping the Metabolic Networks of Tumor Cells and Cancer-Associated Fibroblasts

**DOI:** 10.3390/cells10020304

**Published:** 2021-02-02

**Authors:** Jessica Karta, Ysaline Bossicard, Konstantinos Kotzamanis, Helmut Dolznig, Elisabeth Letellier

**Affiliations:** 1 Molecular Disease Mechanisms Group, Department of Life Sciences and Medicine, Faculty of Science, Technology and Medicine, University of Luxembourg, 6 avenue du Swing, L-4367 Belval, Luxembourg; jessica.karta@uni.lu (J.K.); ysaline.bossicard@uni.lu (Y.B.); Kostas.Kotzamanis@uni.lu (K.K.); 2 Tumor Stroma Interaction Group, Institute of Medical Genetics, Medical University of Vienna, Währinger Strasse 10, 1090 Vienna, Austria; helmut.dolznig@meduniwien.ac.at

**Keywords:** cancer, cancer-associated fibroblasts (CAFs), CAF-tumor cross-talk, tumor metabolism, metabolomics’ measurement techniques, in silico modeling, personalized metabolic drugs

## Abstract

Metabolism is considered to be the core of all cellular activity. Thus, extensive studies of metabolic processes are ongoing in various fields of biology, including cancer research. Cancer cells are known to adapt their metabolism to sustain high proliferation rates and survive in unfavorable environments with low oxygen and nutrient concentrations. Hence, targeting cancer cell metabolism is a promising therapeutic strategy in cancer research. However, cancers consist not only of genetically altered tumor cells but are interwoven with endothelial cells, immune cells and fibroblasts, which together with the extracellular matrix (ECM) constitute the tumor microenvironment (TME). Cancer-associated fibroblasts (CAFs), which are linked to poor prognosis in different cancer types, are one important component of the TME. CAFs play a significant role in reprogramming the metabolic landscape of tumor cells, but how, and in what manner, this interaction takes place remains rather unclear. This review aims to highlight the metabolic landscape of tumor cells and CAFs, including their recently identified subtypes, in different tumor types. In addition, we discuss various in vitro and in vivo metabolic techniques as well as different in silico computational tools that can be used to identify and characterize CAF–tumor cell interactions. Finally, we provide our view on how mapping the complex metabolic networks of stromal-tumor metabolism will help in finding novel metabolic targets for cancer treatment.

## 1. Introduction

### 1.1. CAFs as the Epitome of Tumor Metabolism

Max Borst first noted the importance of the tumor microenvironment (TME) on cancer progression in 1902 [1]. Today, it is well acknowledged that the tumor mass includes not only a highly heterogenous cancer cell population but also various types of resident and infiltrating host cells. Cancer-associated fibroblasts (CAFs) are a major component of the TME. CAFs are fibroblasts that display an activated phenotype: they tend to be larger than their normal counterparts, are spindle-shaped and show the presence of stress fibers. This phenotype is transiently observed in normal fibroblasts during wound healing. In contrast, CAFs seem to be constantly activated and unable to revert to a quiescent phenotype [2]. This observation contributed to Harold Dvorak’s definition of cancer as “the wound that does not heal” [3]. Additionally, the CAF genome was reported to be subject to epigenetic reprogramming whereas most studies have not found mutations in CAFs [4].

CAFs communicate with cancer cells in various ways in supporting tumorigenesis. These include signaling molecules, secretion of growth factors, interleukins and metabolite exchanges [5]. CAFs secrete extracellular matrix components such as collagen and laminins, and produce a plethora of cytokines and chemokines (e.g., interferon-γ, stromal cell-derived factor-1 (SDF-1) commonly known as C-X-C Motif Chemokine Ligand 12 (CXCL12), TNF-α and several interleukins), as well as growth factors (e.g., nodal, transforming growth factor β (TGFβ), fibroblast growth factor (FGF)) [6,7,8]. CAFs also secrete pro-angiogenic factors like vascular endothelial factor (VEGF) and endothelial growth factor (EGF). Most of these factors display pro-tumorigenic effects leading to disease progression. Consequently, the past two decades witnessed the development of novel approaches, which focused on targeting stromal cells. Significant reviews have extensively described the stroma–CAFs–cancer cell interactions [9,10,11,12,13,14,15,16]. Nevertheless, metabolic reprogramming by these tumor–stroma interactions was barely addressed so far and rarely considered as a novel therapeutic avenue. 

Thus, we focus in this review on our current understanding of the mechanisms underlying cancer cell–CAF metabolic interactions, which need further investigation in order to develop novel therapies. We discuss in detail, the activation of different metabolic pathways, which results from the interaction between tumor cells and CAFs. Distinct metabolic analyses on the tumor-CAF crosstalk, covering in vitro, in vivo and in silico modelling approaches, are described and critically evaluated. We present the idea of mapping the complex metabolic landscape of tumor cells and CAFs in order to reach mechanistic insights, by the integration of multi-omics data into context-specific metabolic models. Finally, we discuss the future development of therapeutic strategies considering metabolic targets identified in CAFs.

### 1.2. CAF Heterogeneity: The Different CAF Subtypes

**Figure 1:**
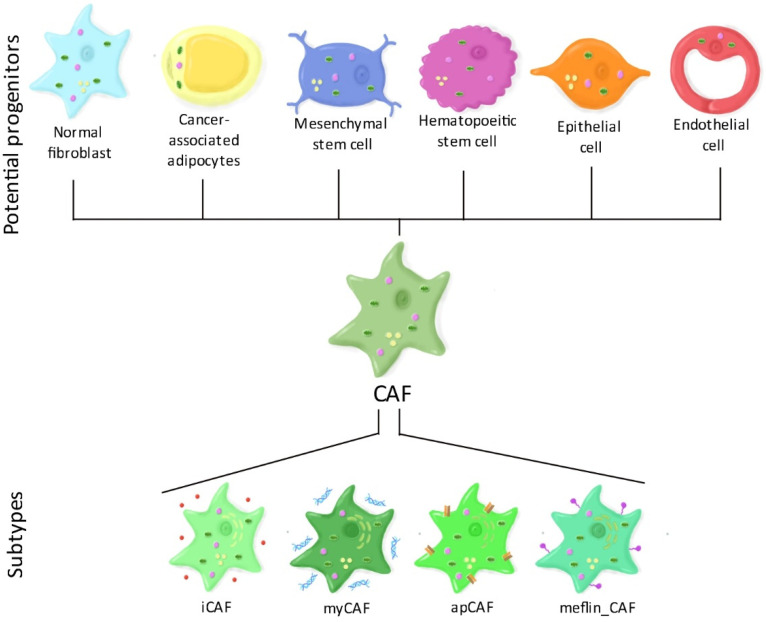
CAF heterogeneity. The origin of cancer-associated fibroblasts (CAFs) remains unclear. Several potential progenitors have been identified including normal fibroblasts, cancer-associated adipocytes, mesenchymal or hematopoietic stem cells, epithelial cells, and endothelial cells. Such heterogeneity in progenitors suggest that CAFs consist of several populations. Today, two principal CAF subtypes have been established: Inflammatory fibroblasts (iCAF) that secrete high levels of cytokines, and myofibroblasts (myCAF) that secrete extracellular matrix components. Additionally, new putative subtypes have been suggested such as antigen-presenting CAFs (apCAF) that express major histocompatibility complex (MHC) class II genes and could be responsible for CD4+ T-cells deactivation, and meflin-expressing CAFs (meflin-CAF), which were found to reduce tumor progression.

There are several theories regarding the origins of CAFs (Figure 1). A first theory suggests that CAFs may derive from the reprograming (and also metabolic rewiring) of normal resident fibroblasts, triggered by bone marrow-derived mesenchymal stem cells [17] or from cancer cells [18,19]. A second theory proposes that CAFs may derive, at least partially, from cancer-associated adipocytes or their progenitor stem cells when exposed to cancer cells [20]. Third, CAFs could originate from both mesenchymal and hematopoietic stem cells, however, the process of this differentiation is still under study [21]. A fourth possibility would be that CAFs originate from epithelial cells through a process called “epithelial to mesenchymal transition” or, similarly, from endothelial cells through “endothelial to mesenchymal transition” [22].

Such heterogeneity in their origin suggests that CAFs consist of several subpopulations. Alternatively, it is possible that CAFs respond to specific signals at different topological sites within the TME, leading to different activation states of CAFs and thus contributing to CAF heterogeneity. Today, four putative CAF subtypes have been identified, especially in pancreatic cancer. Myofibroblasts (myCAFs) express high levels of α-SMA and contractile proteins. They are mostly involved in extracellular matrix remodeling, muscle contraction and focal adhesion. Inflammatory fibroblasts (iCAFs) secrete high amounts of cytokines such as IL-6 and IL-8. They also strongly synthesize matrix proteins, especially hyaluronan and are involved in various inflammatory pathways such as nuclear factor kappa-light-chain-enhancer of activated B cells (NF-κB) and janus kinase/signal transducers and activators of transcription (JAK/STAT) signaling [23]. Interestingly, myCAFs and iCAFs have different effects on the response to immunotherapy. myCAFs were found to be responsible for primary immunosuppression by increasing the infiltration of regulatory T-cells and reducing effector T-cell infiltration, whereas iCAFs were associated with an immunocompetent environment in breast cancer [24]. Recently, new putative subtypes have been suggested. Antigen-presenting CAFs (apCAFs) express major histocompatibility complex (MHC) class II genes and could be responsible for CD4+ T-cells deactivation [25]. Finally, CAFs that express meflin (meflin_CAFs), a membrane-anchored protein were found to decrease tumor progression in pancreatic cancer [26].

Recently, Kieffer and colleagues suggested that in breast and ovarian cancer, CAFs can also be separated into four different subsets according to their location and the expression of specific markers including FAP, α-SMA and CD29 [24]. While two of these subsets (2 and 3) are also found in healthy tissues, subset 1 and 4 are specific to tumors and lymph nodes and are involved in the metastatic process. The subset 1 is additionally involved in immunotherapy resistance [24,27,28]. 

However, the impact of CAF heterogeneity on tumor progression remains widely unknown. It is tempting to speculate that metabolic modelling coupled with single cell RNA (scRNA) sequencing will allow shedding light on the metabolic differences between the CAF subtypes.

## 2. Tumor Cell/CAF Interaction-Driven Metabolic Rewiring in Cancer

CAFs have been considered to act as major regulators in shaping tumor metabolism especially through the dysregulation of several metabolic pathways including glucose, amino acid and lipid metabolism [27,28]. The orchestration of these metabolic switches is believed to shape distinct CAF behavior and change tumor cell behavior by these CAFs. We have summarized the studies on the interplay between CAFs and tumor cells in regard to metabolic reprogramming in Table 1.

### 2.1. Glucose Metabolism and Other Sugar Metabolism

In the presence of O_2_, cells utilize glycolysis to catabolize glucose into pyruvate. The generated pyruvate enters the tricarboxylic acid (TCA) cycle, where it is further oxidized and used to produce energy through oxidative phosphorylation (OXPHOS). In anaerobic conditions, cells oxidize glucose by converting it to lactate, due to the lack of O_2,_ they are unable to utilize OXPHOS. This is a very inefficient process and cells normally only convert a very small amount of glucose to lactate. However, in a phenomenon first identified in the early 20th century by Otto Warburg, cancer cells in culture appear to be reducing a significant percentage of glucose to lactate even in the presence of O_2_. This phenomenon, known as the “Warburg Effect” [46], has been extensively studied and believed to hold true in in vitro models [47,48]. In line, some cancer types in vivo, such as renal cell carcinomas, clearly rely on aerobic glycolysis to generate energy [49]. However, recent findings have demonstrated that this is not a common model applicable to all cancer types in vivo. There is evidence, by stable isotope tracing and mass spectrometry, that some types of lung, liver and brain tumors display high levels of complete glucose oxidation in vivo [50]. Therefore, these studies suggest that, in vivo, the origin of the tumor and the environmental niche as well as the metastatic niche play a crucial role in shaping the metabolic profile of the tumor cells, challenging the Warburg effect model to be of general validity.

Even further complicating the metabolic routes in cancers, in the reverse Warburg model [51], cancer cells highjack CAFs and reprogram their metabolism to adopt an aerobic glycolysis, which is in part triggered by the increasing production of reactive oxygen species (ROS) by neighboring cancer cells. CAFs consequently secrete metabolites such as pyruvate and lactate, which are taken up by cancer cells and used to support their metabolic needs as alternative carbon sources. In this phenomenon mono-carboxylate transporters (MCTs), both on CAFs and cancer cells, facilitate the metabolite exchange between the two cells types [34]. CAFs might thus become continuously exhausted and replaced by novel recruited fibroblasts. This might explain the rapid loss of the human tumor stromal CAFs and their replacement by their murine counterparts in patient derived xenograft models [52].

CAFs also have the ability to reshape and change the extracellular matrix. In cancer, it is well-established that during cancer progression tissue fibrosis and increased ECM stiffness can appear due to changes in the stroma cell phenotype. The interlinked relationships between ECM remodeling, CAF and cancer progression were demonstrated by Bertero et al. in 2019 [53]. The authors found that stiffening of the ECM by CAFs activated the Yes-associated protein 1 (YAP) and Transcriptional coactivator with PDZ-binding motif (TAZ) transcriptional programs, increasing transcription of glutaminase synthase (GLS), lactate dehydrogenase A (LDHA) and aspartate/glutamate transporter SLC1A3 genes. These changes increased metabolite exchange that mutualistically sustained pro-tumor activities in both the cancer cells and the CAF compartment [53]. In hepatocellular carcinoma, induced ECM stiffness activated YAP downstream of c-Jun N-terminal kinases (JNK) and p38. In turn YAP activation increased the glycolytic pathway and migration of cancer cells [54]. YAP/TAZ has also been found to promote cancer cell proliferation by upregulating deoxynucleotide synthesis and inhibiting RAS-induced senescence in cells. However, YAP was inhibited by plating cells on soft substrates which induced expression of senescence markers [55]. These changes can also affect metabolism in cancer cells directly. Plating triple-negative breast cancer cells on denser collagen substrates has been shown to cause a shift towards aerobic glycolysis [56]. In a mechanistically more detailed study, Park et al. in 2019 demonstrated that F-actin regulated degradation of phosphofructokinase (PFK) via ligase tripartite motif containing-21 (TRIM21) [57]. Under mechanical stress TRIM21 was bound to the F-actin bundles of the cytoskeleton and thus PFK degradation was prevented. Transformed cells bypassed this form of regulation by having thick bundles of F-actin that did not respond to mechanical cues. These data indicate that ECM remodeling is directly influenced by CAFs and reciprocally also influences CAFs in cancer. Moreover, these changes in ECM influence directly or indirectly the metabolic phenotype and progression of tumors. 

### 2.2. Amino Acid Metabolism

Human proteins are composed by 20 different proteinogenic amino acids, which are divided into two different groups: the essential amino acids (EAAs: histidine, isoleucine, leucine, lysine, methionine, phenylalanine, threonine, tryptophan and valine) and the non-essential amino acids (NEAAs: alanine, aspartate, asparagine, arginine, cysteine, glutamate, glutamine, glycine, proline, serine and tyrosine) [58]. In cancer, amino acids, apart from their normal role as protein building blocks, can serve as alternative energy source to fuel the TCA cycle and can regulate redox status as well as antioxidant defense [58,59]_._ They can also act as substrates for post-translational and epigenetic transformation [58,59]. These versatile aspects of amino acids led to a surge of interest in further investigating their mechanistic roles in tumor metabolism.

One of the key amino acids, which is abundant in human plasma, is glutamine (Gln). Gln acts as an important precursor for the synthesis of proteins, nucleotides, fatty acids and other critical molecules [60,61]. There are extensive studies on the role of glutamine in regulating cancer cell metabolism [43,47,48]. Several of these have reported crucial roles of Gln metabolism in the interaction between CAFs and tumor cells (Table 1). In ovarian cancer, CAFs were shown to generate high levels of Gln by glutamine synthetase (GS). In this cancer, CAF-derived Gln was exported to the tumor cells and converted to glutamate by the enzyme glutaminase. This further supported tumor cell growth by anaplerosis (replenishment process of metabolic pathway intermediates) of the TCA [38]. Indeed, co-inhibition of glutamine synthetase (GLUL) in CAFs and glutaminase (GLS) in ovarian cancer cells abrogated cancer cell growth better than each individual treatment [38]. Furthermore, in a recent study, it was found that CAFs migrate from the glutamine depleted core of tumors towards more glutamine rich areas. This migration based on a glutamine gradient was dependent on protein kinase B (ATK2) and allowed tumor cells to escape the orginal tumor site [37].

Additionally, CAF-derived Gln has been reported to activate a process called reductive carboxylation in pancreatic ductal adenocarcinoma (PDAC) [36]. Reductive carboxylation of glutamine is a thoroughly studied metabolic route in which glutamine is converted to α-ketoglutarate (α-KG) to enter the TCA cycle. Then, in a reversal of the TCA cycle, α-KG is converted to citrate, which is finally exported to the cytoplasm where it contributes to fatty acid biosynthesis. This glutamine-dependent metabolic reprogramming was induced by the transfer of CAF-derived exosomes (CDEs containing glutamine) into PDAC cells and was shown to be independent of KRAS activation [36].

Further research suggests that CAFs can transfer aspartate to cancer cells via the SLC1A3 transporter (also known as excitatory amino acid transporter 1 [EAAT1]), to facilitate nucleotide synthesis in the tumor cells [53], whereas glutamine-derived glutamate from cancer cells is taken up by CAFs through the same transporter.

Pancreatic stellate cells (PSCs), one of the best studied CAF subtypes in PDAC, can be reprogrammed to secrete alanine through activation of autophagy. The secreted alanine is taken up by the tumor cells and converted to pyruvate which fuels the TCA. This further allows PDAC cells to divert glucose carbon atoms into serine/glycine metabolism for sustaining their proliferation. The dysregulation of tryptophan metabolism due to tumor–CAF interaction has also been reported (Table 1). Yet, there are still many amino acids which have not been explored in the metabolic crosstalk between CAF and tumor cells. Obviously, further research is needed to dissect the metabolic function of other amino acids within tumor–CAFs interactions.

### 2.3. Lipid Metabolism

Lipids are macromolecules that are soluble in non-polar solvents. In biology they include substances such as fats, waxes, oils, vitamins and steroid hormones. Fatty acids (FAs) are the building blocks of various lipids. Lipids and FAs have main functions in cells: (i) they build up the structural components of cell membranes which mainly consist of glycerophospholipids and sphingolipids (ii) they provide energy storage in the form of triglycerides and (iii) they work as signaling molecules [62]. Given these functions, it is not surprising that increased de novo FA synthesis has been observed in various types of cancer in order to support rapid tumor cell growth [63,64].

Limited studies have investigated how the dysregulation of lipid metabolism in CAFs affects tumorigenesis. In an early study in 2013 by Kamphorst et al. the authors were able to show that hypoxic cancer cells scavenge FA from lysophospholipids [65]. In 2019, Auciello et al. further demonstrated that CAFs secrete lysophospholipids, particularly lysophosphatidylcholine, to promote tumor proliferation and metastatic processes in PDAC [19]. In a similar study, lysophosphatidic acid from ovarian cancer cells induced a CAF phenotype in peritumoral fibroblasts, indicating again how the crosstalk between CAFs and cancer cells is mutualistic in nature [44]. Finally, a recent study indicates that lipids secreted by CAFs are taken up by colorectal cancer (CRC) cells and that deletion of the fatty acid synthase (FASN) or the inhibition of FA uptake in CAFs reduced CRC cell migration [45].

Recently, more attention has come to an understanding on how mechanical forces in the TME can regulate lipid metabolism. In 2019, Romani et al. demonstrated that a soft ECM microenvironment increased lipid and cholesterol synthesis [66]. Mechanistically it was identified that reduced mechanical stress on the Golgi apparatus led to sterol regulatory-element binding proteins (SREBP) activation and translocation to the nucleus, where it induced activation of lipid synthesis [66]. Conversely, in 2018 Boulter et al. uncovered that genetic deletion of the amino acid transporter and integrin coreceptor CD98hc (SLC3A2) indirectly influenced mechanosensing of integrins by impairment in sphingolipid metabolism [67].

### 2.4. Immune Modulation by CAF-Derived Metabolism

CAFs have been hypothesized to play an important role in immune evasion (for a more in depth review please refer to [68]). CAF-secreted metabolites, such as lactate, can actively participate in an immunosuppressive TME. Several studies have attempted to further clarify the role of CAF-derived metabolites in modulating anti-tumor immunity. In breast cancer, CAF-secreted kynurenine, a tryptophan metabolite, was found to promote E-cadherin/Aryl hydrocarbon receptor (AhR)/S-phase kinase-associated protein 2 (Skp2) complex, leading to E-cadherin degradation, which supported cancer cell invasion [40]. Indoleamine 2,3-dioxygenase (IDO), the key enzyme in the degradation process of tryptophan to kynurenine, was found to be expressed by CAFs. The STAT3-dependent release of prostaglandin E2 by cancer cells triggered the upregulation of IDO in CAFs. Moreover, the co-expression of IDO in stromal fibroblasts and cyclooxygenase (COX2) in breast tumors was correlated with poor patient survival and metastasis spreading. CAFs-derived IDO can also induce T cell anergy and inhibit CD8^+^ cytotoxic activity [69]. Similarly, the upregulation of galectin-1 in lung cancer cells induced CAFs to overexpress tryptophan 2,3-dioxygenase (TDO2) and enabled CAFs to secrete kynurenine [39]. This kynurenine/TDO2 signaling was found to promote cancer growth and invasion, while suppressing the differentiation of dendritic cells through the AKT/cAMP response element-binding protein (CREB)/ WNK Lysine Deficient Protein Kinase 1 (WNK1) axis. The production of arginase II (ARG 2) by CAFs was also reported to hamper anti-tumor T cell functions [70].

Moreover, a reciprocal interaction between CAFs and neutrophils has been described [71]. Activated neutrophils are high producers of ROS [72]. Using a lymphoma mouse model, a study showed that tumor cells educated CAFs to enhance the recruitment of CD11b^+^Ly6G^+^ neutrophils via the CCL2-CCR-2 axis and accelerated tumor growth [73]. Activated neutrophils can also stimulate the transformation of MSCs into highly FAP expressing CAFs and promote the metastasis of gastric cancer cells via IL6/STAT3 axis [74]. In hepatocellular carcinoma, α-SMA^+^ CAFs are found to produce IL-6 for recruiting neutrophils via the activation of STAT3 and c-Jun kinase-programmed cell death ligand 1 (STAT3-PDL1) [75]. This signaling cascade induces neutrophil apoptosis and fosters tumor progression. Immunosuppressive roles of CAFs on macrophages and CD8^+^ T cells have also been shown to be ROS-dependent [76,77,78]. CAFs can additionally lead to extensive reorganization of the mitochondrial metabolism in prostate cancer in a STIRT1/PGC-1a dependent manner [79]. This leads to extensive mitochondria-generated ROS (mtROS) generation that supports pro-invasive features in prostate cancer cells.

## 3. CAFs and Reactive Oxygen Species

Reactive oxygen species (ROS) consist of radical and non-radical oxygen species generated by the partial reduction of oxygen. Despite the well-known ROS-driven damaging effects, ROS, and especially mitochondria-generated ROS (mtROS), have been shown to play a key role in diverse cell functions of different cell types [80,81]. In cancer, ROS generation is greatly enhanced, especially in some cancer types with known mutations to mitochondrial enzymes. ROS can activate cell survival through the activation of different signaling cascades including the JNK, the MAPK/ERK1/2, the PI3K/Akt, the p38 MAPK, the NF-kB, as well as the activation of transcription factors, such as HIF1a and NRF2 [82,83,84,85].

CAFs produce ROS in their permanent activated state. Oxidative stress in fibroblasts during chronic inflammation and in cancers, which is mediated by ROS, can induce genomic instability of the adjacent epithelial cells and thus, contribute to tumor initiation or drive tumor progression [83]. ROS mediates the loss of stromal Cav-1, which is associated with a more aggressive phenotype in pancreatic [86] and breast cancers [87]. Cav-1, a structural component of caveolae and signaling regulator, which is composed by sphingolipids and cholesterol, negatively regulates NADPH oxidase, a major ROS producer, through different pathways [88]. Stroma remodeling potentially occurs due to the high level of ROS that lead to the differentiation of normal fibroblast into activated, pro-tumorigenic myofibroblasts [89]. In support of this hypothesis, the treatment of CAF-conditioned media or exogenous ROS on normal fibroblasts (NFs) led NFs to adopt oxidative CAF-like features [90]. TGF-β signaling has been described as an important factor in the conversion from NF to CAF [91,92]. Despite these findings, other studies indicated that TGF-β signaling ablation in stromal fibroblasts still resulted in tumor growth and progression [93,94]. In 2017, Chan et al. were able to demonstrate that impaired TGF-β signaling leads to a decreased antioxidant enzyme glutathione peroxidase (GPx1) consequently increasing ROS levels and fueling tumor growth [95]. Apart from inducing ROS, CAFs can also help alleviate ROS induced oxidative stress in cancer cells by enhancing glutathione production in response to platinum-based chemotherapies [96]. Hence, ROS are key regulators in the tumor–CAF metabolic interactions, which strongly influence the behaviors and functions of both CAFs and tumor cells.

## 4. Metabolic Approaches to Study the Cross-Talks between CAFs and Tumor Cells

The proper analysis of metabolism in cell-cell interaction is dependent on specific analytic techniques to measure the metabolome, which represents the pool of metabolites from diverse array of metabolic activities [97,98]. Here we discuss the different methods that allow us deciphering the metabolic crosstalk between different cells, e.g., of CAFs and tumor cells.

### 4.1. Mass Spectrometry-Based Metabolomics

Generally, mass spectrometry (MS) is the leading technology used to identify metabolites and quantify their concentration [99]. It allows obtaining both quantitative and qualitative information about the intracellular or extracellular metabolome composition of a given biological sample [97]. MS is commonly coupled with gas chromatography (GC) or liquid chromatography (LC), to allow pre-separation of metabolites. GS tandem MS requires the derivatization of metabolites and their conversion to the air phase. This limits the number of molecules that can reliably be measured. GC-MS, however, is an ideal technique for analyzing non-polar, volatile and non-volatile molecules of small molecular weight [100]. Conversely, LS-MS is well-suited to analyze thermally unstable molecules and requires a less extensive extraction process. These metabolites are then loaded on the MS to identify and decode the metabolic profiles of cells or tissues. This kind of metabolite analysis can be used for a targeted (with pre-defined or selected metabolites targets) and a non-targeted metabolomics approach.

In the studies of CAF and tumor cell interactions, the use of LC-MS is more popular compared with GC-MS (Table 1). This is probably due to the broader coverage of metabolite read-outs and a simpler methodical setup in LC-MS compared to GC-MS. A few studies have also used superior versions of the conventional LC, namely ultra-performance liquid chromatography (UPLC) or high-performance liquid chromatography (HPLC) to assess metabolism in tumor–CAF interaction. Both allow the separation of smaller particle sizes (<2 µm for UPLC) and provide a higher speed, resolution, and sensitivity [101]. However, MS is mostly used to analyze in vitro cultures and ex vivo tumor tissues. More applications of those techniques are needed to further explore CAF and tumor metabolic interactions under in vitro and/or in vivo settings. In the future, they will be particularly useful in delineating the role of small extracellular vesicles, which are derived from CAFs, and are capable of driving tumor metabolic rewiring. Moreover, integrating the MS-measurements with other metabolic approaches such as stable isotope labelling and seahorse assays seems an ideal way to decipher CAF and tumor cell metabolic interactions.

### 4.2. Metabolic Flux Analysis

While measuring the level of cellular metabolites provides crucial information, it is equally important to determine the pathways that are linked to them and how they interact. On its own, a drop in the levels of a metabolite can either point towards the reduction of the synthesis or the increased consumption of this metabolite via another pathway. One of the metabolic techniques used to determine the pathways that contribute to metabolite secretion is known as ‘metabolic flux analysis’ (MFA). MFA uses nutrients, such as glucose, amino acids, or lipids, which are labelled with the stable isotopes ^13^C or ^14^N, as the basis for metabolites’ identification and quantification [102]. Mass spectrometry is commonly used to analyze the labeled samples. Primarily, ^13^C-MFA aims to produce a quantitative cellular metabolic map by assigning the values of fluxes to the reactions in the network and by using confidence interval for every predicted flux [103].

Although MFA has been used to elucidate the metabolic changes that take place in cancer cells and their microenvironment in vitro, it is equally important to perform ^13^C-MFA under in vivo settings, which has been addressed recently [101,104]. In fact, the variations in experimental set-up between different in vitro studies on CAF–tumor interaction has also raised concerns about the most reliable workflow to use. Thus, its usage for understanding CAF–tumor cell interactions is still limited (Table 1), particularly under the in vivo or ex vivo settings. However, in vivo flux analysis provides a better comprehensive view of the metabolism at the cellular and whole-organism level which can complement in vitro approaches [105,106]. In particular, it will help to understand the nutrient exchange between CAFs and the other components of the TME, such as immune cells or pericytes, which are known to impact tumorigenesis. Furthermore, by using ^13^C-MFA in vivo, it will be possible to understand the contribution of each metabolic pathway to the generation of CAF-secreted oncogenic metabolites in a physiological relevant context. Accordingly, the integration of MFA with other computational approaches will lead to a better understanding of the CAF–tumor interaction-driven cancer metabolic rewiring in the future.

### 4.3. Seahorse Extracellular Flux

The Agilent Seahorse XF Analyzer is a powerful tool that can offer a rapid insight into the metabolic state of the cells. Seahorse protocols use a combination of metabolic inhibitors to quantify the rate of glycolysis and/or OXPHOS, via the measurement of extracellular acidification rate (ECAR) and oxygen consumption rate (OCR). By understanding the cellular responses to metabolic drugs, the different respiratory phases can be defined and the cellular bioenergetics status of the cells can be inferred [107].

In ECAR measurement, cells are stimulated with 2,4-Dinitrophenol (2,4-DNP, an oxidative phosphorylation inhibitor) and 2-Deoxy-D-Glucose (2-DG, a hexokinase inhibitor). 2-4 DNP induces maximal glycolytic capacity, whereas 2-DG is a glucose analog that completely shuts glycolysis. For measuring OCR, cells are exposed to mitochondria perturbing reagents. Oligomycin, carbonyl cyanide-4-(trifluoromethoxy)phenylhydrazone (FCCP) and rotenone coupled with antimycin are added to the cells in a sequential manner to induce changes in mitochondrial activity. Oligomycin blocks the ATP synthase (complex V) and suppresses OCR. FCCP is a mitochondrial uncoupler which disrupts the proton gradient and mitochondrial membrane potential, and drives OCR to a maximal level, which is then reduced to a minimal level by antimycin A and rotenone (complex III and complex I inhibitor), finally disrupting mitochondrial respiration. The administration of these drugs at different time-points causes the fluctuation in the OCR level. These data can then be used to calculate basal respiration, ATP-linked respiration, proton leak, maximal respiration capacity, reserve capacity and non-mitochondrial respiration [108].

An indirect in vitro study set-up using the conditioned media, of either the tumor cells or the CAFs, is commonly used to study CAF–tumor cell interaction with Seahorse (Table 1). This is obviously due to the simplicity of such a set-up. In addition, most studies have only been performed on a 2D cell culture monolayer so far. However, it is commonly accepted that 3D cell cultures better recapitulate the physiological parameters of a tumor; they have been described to better mimic cell–cell interaction and environmental factors (gradient of oxygen and nutrient supply) compared to their 2D counterparts [109,110].

In a more advanced setup, Demircioglu et al., 2020 have used Seahorse to analyze isolated cells from ex vivo tumor samples [29]. Such an approach may give deeper insights into the physiological and metabolic state of a tumor. Yet, the prior tissue processing steps to isolate the cells need to be considered as these may significantly alter the initial tumor metabolic state.

Thus, further explorations on 3D cultures and direct co-culture settings with Seahorse, in combination with other metabolic measurements (such as stable isotope tracing and non-destructive metabolic imaging approaches), are definitively warranted in order to gain greater mechanistic insights into CAF–tumor cell interaction.

### 4.4. Computational Approaches to Unravel Tumor-CAF Metabolic Reprogramming

The increasing use of modern analytical techniques has helped characterizing tumor metabolism. However, the heterogeneity of the tumor metabolic rewiring is still not yet fully understood. Studies often only touch upon the phenotypic analysis without providing an in-depth mechanism. This is mostly due to the technical challenges and experimental limitations in the field of metabolomics. While metabolomics studies provide powerful insights into the cellular metabolic status of cells, the information regarding the cause or effect of metabolite changes is still missing, hence limiting our understanding of the underlying mechanisms. The cell’s metabolome constitutes the amplified and integrated signals from various levels, either at the transcriptional level or post-translational level [111]. Therefore, a multi-omics approach (proteomics, transcriptomics and metabolomics) may provide a bigger picture to identify the key molecular regulators of a complex disease, like cancer. Further integration of ‘omics’ data to build personalized in silico models (i.e., models which represent each patient individually) could shed light on the metabolic heterogeneity and the regulatory interactions between different classes of biomolecules (genes, proteins and metabolites), especially in CAF subpopulations which are continuously being identified.

The principle of network modelling is to present high-throughput data in a robust and predictive way [112]. Metabolic models are computational tools showing the metabolites as a set of nodes and the enzymatic conversion from one metabolite to another as edges [113]. The main advantage of computational metabolic networks is the possibility to narrow down the number of potential targets to be validated in vitro, therefore saving time and money. They also allow studying the complex interactions between the tumor and its microenvironment on multiple scales and generating patient-specific models [114]. As such, they represent important tools to improve drug testing, thereby making treatments more accurate and beneficial to the patients [115].

Genome-scale metabolic reconstructions, such as Recon2 [116], iHSA [117], or Human1 [118], consist of a library of all the possible reactions that can occur in an organism, as well as a set of genes that control the enzymes and transporters that allow the reactions to take place [119]. There are two types of metabolic models. Kinetic models rely on ordinary differential equations and kinetic rates. Their applicability is, however, limited, due to a high number of parameters to be determined [120]. Constraint-based models focus on steady states and do not integrate parameters, which allows reconstructing and analyzing larger models. Over the last few years, these models have gained interest to model the metabolic status of a cell [121]. One important advantage of constraint-based modelling is the possibility to integrate multi-omics data, such as transcriptomics [122], proteomics [123] or metabolomics [124], to constrain genome-scale metabolic reconstructions and generate context-specific models [125]. Several algorithms exist that build context-specific models such as iMAT [126], INIT [127], mCADRE [128] and the FASTCORE family [129,130,131]. Finally, in silico gene knock-out can be applied to identify potential drug targets. Gene knock-out consists of blocking all the reactions associated with a specific gene and assesses its effect on the objective function of the model, i.e., biomass production. The genes that significantly reduce growth are considered as essential and, therefore, as potential drug targets [132].

However, modelling a complex system such as the tumor microenvironment remains challenging, mainly due to the difficulty in modelling the interactions between multiple cell types. Indeed, such models already exist for microbial communities [133], however, similar approaches applied to multiple human cell types are still widely unexplored. In 2010, Lewis et al. built a constraint-based model for multiple cell interactions in the brain based on Recon1 and successfully simulated Alzheimer’s disease [134]. In 2015, Capuani et al. reconstructed a small, unconstrained model (75 reactions) for the lactate shuttle between CAFs and tumor cells [135]. More recently, in 2018 Shan et al. modeled the impact of the tumor microenvironment on the Warburg effect and glutamine addiction in cancer cells and found that the reverse Warburg effect provided growth advantage to the tumors originating from deep tissues [136]. Finally, in 2019 Damiani et al. developed a new algorithm (single-cell flux balance analysis) that allows integrating single-cell RNA-seq data into models of breast and lung cancer. This method allowed them to model an heterogeneous cancer cell population, to identify cancer cell subpopulations based on their growth rate and enabled to represent metabolite exchange between different cell types [52,137].

These first studies strongly demonstrate that there is no doubt that constrain-based modelling combined with single-cell sequencing will become one of the most important tools to study tumor–microenvironment interactions. Additionally, many efforts are now directed towards the integration of signaling data into metabolic models.

## 5. Concluding Remarks

Based on their predominantly tumor promoting function, targeting CAFs has become one of the promising alternative therapeutic approaches in cancer. The relatively stable genomic status, which may minimize resistance, gives a major advantage to CAF-targeted therapies [138]. CAFs can also limit the anti-tumor immunity by either reducing immune cell infiltration or increasing an immunosuppressive state, or a combination of both. These interactions are suggested to impair the response to current immunotherapies in different cancer types.

**Figure 2:**
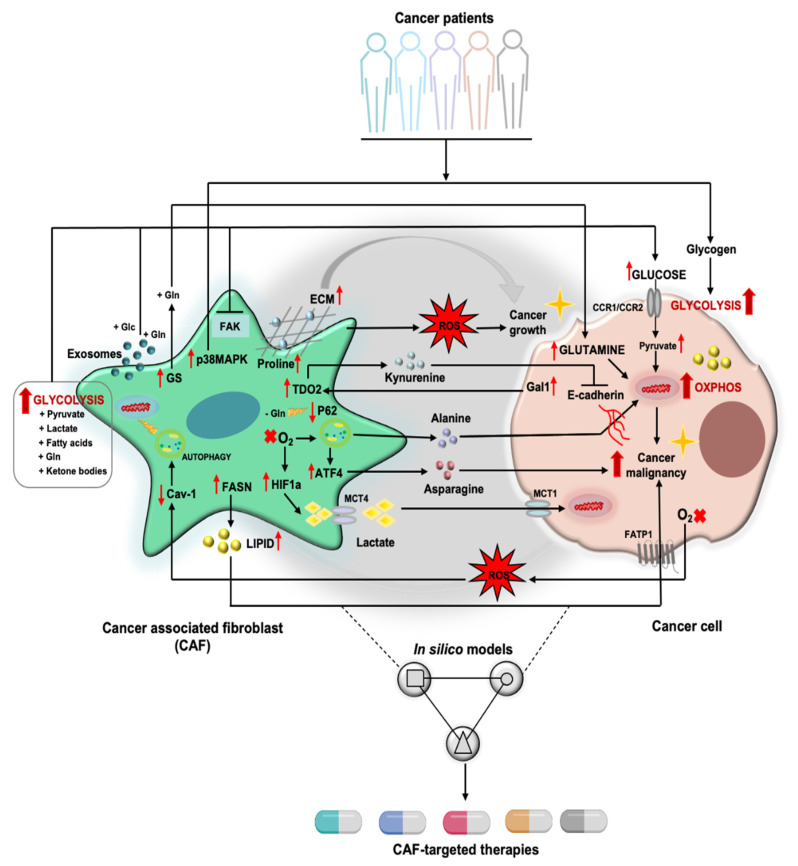
Future perspective on mapping the metabolic CAF–tumor cells interactions. The interaction of CAF and cancer cells lead to metabolic reprogramming in tumors, majorly in glucose, amino acids and lipid metabolism. Reprogrammed CAFs increase several amino acids (glutamine, kynurenine, alanine, asparagine, proline), lipid (LPC) and lactate secretions which fuels malignant glucose cancer metabolism, supporting tumor growth. CAFs-derived exosomes also contribute in driving carcinogenesis by supplying glucose and glutamine to cancer cells. ROS, produced by CAFs or tumor cells, become an important mediator in CAFs–tumor cells’ metabolic interactions further supporting cancer malignancy. Yet, there is no therapy that specifically targets CAFs’ metabolism most probably due to the high CAF heterogeneity. In silico approaches may help in unravelling the mechanistic insights of CAF-tumor crosstalk, ultimately identifying tailored CAF-targeted therapies. Glc (Glucose), Gln (Glutamine), GS (Glutamine synthetase), FAK (Focal adhesion kinase), MAPK (mitogen-activated protein kinases), ECM (Extracellular matrix), ROS (Reactive oxygen species), CCR (Chemokine receptor), TDO2 (Tryptophan 2,3-Dioxygenase), Cav-1 (Caveolin-1), FASN (Fatty acid synthase), LPC (Lysophophatidylcholine), HIF1a (Hypoxia-inducible factor 1-alpha), ATF4 (Activating Transcription Factor 4), MCT (Monocarboxylate Transporter), FATP1 (Fatty acid transport protein 1).

In the light of the direct tumor promoting activity and the immunomodulatory capacity of CAFs, researchers have focused over the past years on testing inhibitors and antibodies that would specifically target CAFs [10]. These drugs aim at targeting either CAFs directly or their secreted molecules, which are known to sustain tumor progression. Another appealing therapeutic strategy is the reprogramming of CAFs into normal fibroblasts or into an antitumorigenic phenotype which suppresses carcinogenesis [10]. However, due to CAF heterogeneity and the varying nature of the TME in different tumor sites, the exact mechanisms behind this regulation remains rather elusive. The high heterogeneity and plasticity of CAFs are believed to contribute to the unsuccessful outcomes observed in clinical trials using CAF-targeted therapies [11,68]. Moreover, drugs that specifically target CAFs metabolism have not been identified so far [139]. Thus, mapping a personalized network with in silico approaches based on an individual patient’s biological information may potentially lead to the identification of better therapeutic strategies (Figure 2). The advancement in experimental approaches will encourage gathering all the necessary information to build such personalized in silico metabolic models in the future. Finally, the integration of all experimental data into these personalized models will allow for better tailoring of metabolic anti-cancer therapies for patients.

## Figures and Tables

**Table 1:** Summary of the CAF-tumor cell crosstalk metabolic studies. Abbreviations: Glucose (Glc), Glutamine (Gln), NA, Not Applicable.

Cancer Type	In Vitro Analysis	In Vivo Analysis	Ex Vivo Analysis	Technical Advantage	Technical Limitation	Ref.
**Glucose metabolism**						
Breast and pancreatic	NA	F-FDG-PET/CT glucose imaging U-13C6-glucose tracing	Seahorse Proteomics and phosphoproteomics	Comprehensive metabolic analyses on ex vivo samples Implementation of in vivo metabolic analysis	Murine material only	Demircioglu et al. 2020 [29]
Mechanistic highlight: FAK-deletion in CAFs induced malignant cell glycolysis and tumor growth via CCR1/CCR2						
Breast	U-13C6-glucose tracing Seahorse Lactate measurement	NA	LC-MS metabolomics	Comprehensive metabolite analyses on in vitro and ex vivo samples Clear schematic overview of U-13C6-Glc tracing experiment	Minimal metabolic analyses of hypoxia within tumor and CAF interactions No metabolic validation in vivo	L.M. Becker et al. 2020 [30]
Mechanistic highlight: Chronic hypoxia induced NFs to adopt a pro-glycolytic CAF phenotype via epigenetic reprogramming, which fuelled cancer cells’ metabolism and their growth.						
Breast	Glut1 cell surface protein, glucose consumption, lactate secretion and intracellular ROS Seahorse	NA	NA	Investigating the role of hypoxia role on patient-derived fibroblast pairs	No metabolic validation in vivo and ex vivo Limited implementation of metabolic analysis	Sun et al. 2019 [31]
Mechanistic highlight: Lactate secreted by hypoxic and pro-glycolytic CAFs was driven by GLUT1 phosphorylation and PKM2 upregulation, and this lactate promoted cancer cell invasion via activated TGFB1/p38 MAPK/MMP2/9 signaling and increased cancer cells mitochondrial activity.						
Ovarian	Seahorse Metabolite profiling Glycogen, ATP/ADP ratio and lactate Glucose-1-Phosphate 2-NBDG glucose uptake Glucose and amino acids (AAs) tracing Glycogen phosphorylase activity assay	NA	NA	Comprehensive in vitro metabolic analyses related to glycogen on human co-cultured samples	No metabolic validation in vivo nor ex vivo	Curtis et al. 2019 [32]
Mechanistic highlight: Glycogen utilization in cancer cells was dependent on p38-alpha MAPK activation in CAFs and this supported their proliferation, invasion and metastasis						
Lymphoma	Intracellular (CE-TOF/MS read out) and extracellular (HPLC) metabolomics Intracellular ROS and pyruvate measurement Calcein-acetoxymethy ester-based metabolite measurement	NA	NA	Comprehensive metabolic analyses on patient-derived materials for both CAF and tumor cells	No metabolic validation in vivo and ex vivo	Sakamoto et al. 2019 [33]
Mechanistic highlight: CAF-secreted pyruvate supported citric acid cycle while inhibited redox regulation to promote cancer cells survival						
Pancreatic	Glucose, glutamine and lactate secretion U-13C6-Glc and U-13C5-Gln tracing Seahorse	NA	NA	Comprehensive metabolic analyses in vitro	No metabolic validation in vivo and ex vivo *•* Analysis limited to CAFs	Knudsen et al. 2016 [34]
Mechanistic highlight: HIF1alpha-driven hypoxic and pro-glycolytic CAFs upregulated carbonic anhydrase IX (CAIX) and MCT4 expressions, secreted lactate and supported cancer invasion						
**Glucose and glutamine metabolism**						
Breast	U-13C6-Glc, U-13C5-Gln, U-13C3-lactate tracing with NMR analysis Glucose, lactate, glutamate, glutamine, ammonium and NO measurements LDH, GDH and GLUL enzymatic activities measurements Seahorse	U-13C6-Glc, U-13C5-Gln, 15N2 tracing via tail-vein with LC-MS analysis	NA	Exploring the role of EV-encapsulated microRNAs (miRNAs) Investigating the metabolic differences between normal and nutrient-deprived conditions Metabolite tracing in vivo	No metabolic analyses on the tumor cells side	Li et al. 2018 [35]
Mechanistic highlight: Breast cancer-secreted extracellular vesicles (EVs) containing miR-105 induced a MYC-dependent pro-glycolysis and pro-glutaminolysis in CAFs under sufficient nutrients. However, in nutrient-deprived conditions, these mir-105-reprogrammed CAFs converted metabolic wastes (i.e., lactic acid and ammonium) into energy-rich metabolites to sustain tumor growth.						
Prostate and pancreatic	U-13C6-Glc and U-13C5-Gln tracing Seahorse Lactate, acetate and mitochondrial membrane potential/TMRM	NA	NA	Exploring the metabolic roles of CAFs-derived exosomes under the co-culture set-up Metabolic analyses on different cancer types	No metabolic validation in vivo and ex vivo	Zhao et al. 2016 [36]
Mechanistic highlight: Under starvation, CAFs-derived exosomes (CDEs) were smuggled in by cancer cells as the required building blocks. This caused a decrease in mitochondrial OXPHOS, while increase in glucose and glutamine tumor cell metabolism, enhancing cancer cells survival via a Kras-independent mechanism.						
Breast and CRC	Invasion and migration Organotypic invasion Directional migration in chemotaxis μ-slides.	Migration	NA	Exploring the roles of metabolite gradient on CAFs and tumor cell migration		Mestre-Farrera et al. 2020 [37]
Mechanistic insight: CAFs are more sensitive to low glutamine levels than their cancer counterparts. As the tumor core is depleted in glutamine, CAFs move towards glutamine rich areas in an ATK2 dependent mechanism. This movement along with the racks that CAFs create allow tumor cells to invade tissues and escape their original site.						
**Amino acids metabolism**						
Ovarian	Transcriptomic Glutamine secretion measurement by UPLC U-13C6-Glc, U-13C5-Gln and U-13C3-lactate tracing	NA	Glucose and lactate measurements	Comprehensive metabolic analyses in vitro and ex vivo	No clear experimental set-up for in vitro co-culture tracing	Yang et al. 2016 [38]
Mechanistic highlight: Under glutamine deprived conditions, CAFs harnessed atypical carbon and nitrogen sources to boost their glutamine production, and support cancer cells proliferation. This relied on the expression of glutamine synthetase (GLUL) in CAFs and glutaminase (GLS) in cancer cells.						
Lung	ELISA	NA	NA	Revealing the mechanistic insights of fibroblasts’ and immune cells’ interactions in cancer	No metabolic validation in vivo and ex vivo	Hsu et al. 2016 [39]
Mechanistic highlight: Tumor-fibroblast interaction induced galactin-1 overexpression in lung cancer which led to TDO2-dependent kynurenine secretion by fibroblasts. Fibroblasts-secreted kynurenine promoted cancer growth, invasion and immunosuppression through the AKT/CREB/WNK1 axis.						
Breast	Metabolite profiling using UPLC	NA	NA	Revealing the mechanistic insights of fibroblast-derived metabolites	No metabolic validation in vivo and ex vivo	Chen et al. 2014 [40]
Mechanistic highlight: IDO-dependent kynurenine secretion by CAFs promoted E-cadherin degradation and increased invasion of cancer cells. PGE2 released by cancer cells promoted the expression of stromal IDO via STAT3/COX2 activation.						
Pancreatic	Seahorse U-13C-alanine, U-13C6-glc, U-13C5-Gln tracing Steady-state untargeted metabolomics	NA	NA	*•* Conducting in vitro metabolic analysis on the co-culture set-up	No metabolic analysis in vivo/ex vivo No clear in vitro experimental set-up for metabolic tracing	Sousa et al. 2016 [41]
Mechanistic highlight: Autophagy dependent-alanine secretion by PSCs became an alternative carbon source for cancer cells. This led to an increase in the OCR of PDAC cells.						
Breast	MS-phosphoproteomics and proteomics PDH measurement U-13C-proline, U-13C5-Gln, U-13C6-glc, 13C3-pyruvate, 13C-citrate and 13C16-palmitate tracing cholesterol (GC-MS) fatty acid (LC-MS)	NA	NA	Comprehensive metabolic analyses on fibroblasts 2D vs. 3D co-cultures settings	No metabolic analysis using the co-culture settings	Kay et al. 2020 [42]
Mechanistic highlight: Proline synthesis in CAFs caused tumor epigenetic reprogramming, which enhanced ECM production and supported tumor growth.						
**Lipid metabolism**						
Pancreatic	Extra- and intra-cellular LC/MS lipidomics U-13C-palmitate and -oleate tracing	NA	Measurement of lysophosphatidic acid (LPA)	Comprehensive lipid analyses on the co-cultured samples Clear set-up for the stable isotope tracing on co-culture	Limited metabolic analysis on the patients-derived materials	Auciello et al. 2019 [19]
Mechanistic highlight: PSC secreted lysophospatidylcholines (LPC) promoted the secretion of oncogenic autotaxin-lysophospatidic acid (LPA), which supported proliferation, migration and AKT activation in PDAC						
Breast	Intracellular lipid detection (Nile Red staining) Fatty acid synthase (FASN) enzymatic activity	NA	NA	Performing imaging analysis on lipid content	Limited metabolic analyses No metabolic analysis in/ex vivo	Coelho et al. 2018 [43]
Mechanistic highlight: Lipids were transferred from CAFs to tumor cells, which was dependent on fatty acid transporter-1 (FATP1), and promoted tumor growth.						
Breast	ELISA Seahorse	NA	NA	Extensive seahorse analysis in investigating the impact of OCC on ovarian fibroblasts	No metabolic analysis in/ex vivo In vitro metabolic analysis only on fibroblasts	Radhakrishnan et al. 2018 [44]
Mechanistic highlight: Under normoxia and hypoxia, the secreted LPA by ovarian cancer cells (OCC) induced pro-glycolytic phenotypes in both ovarian NFs and CAFs. This was due to LPA triggered HIF1 alpha-dependent pseudohypoxic oxidative stress in OCC.						
Colorectal	Lipidomic analysis by UPLC-Q-TOF/MS			Comprehensive in vitro lipidomic analyses	No metabolic analysis in vivo nor ex vivo	Gong et al. 2020 [45]
Mechanistic highlight: FASN-dependent CAFs-secreted lipids were taken up by tumor cells and induced tumor migration capacity.						

## Data Availability

Not applicable.

## Glossary

**Abbreviation**	**Full Text**
α-KG	Alpha-ketoglutarate
apCAF	Antigen-presenting fibroblasts
CAFs	Cancer-associated fibroblasts
CRC	Colorectal cancer
CDEs	CAF-derived exosomes
EAAs	Essential amino acids
ECAR	Extracellular acidification rate
FA	Fatty acids
FASN	Fatty acid synthase
FCCP	(trifluoromethoxy)phenylhydrazone
GC	Gas chromatography
Glc	Glucose
Gln	Glutamine
GS	Glutamine synthetase
HPLC	High performance liquid chromatography
iCAF	Inflammatory fibroblasts
LC	Liquid chromatography
LPC	Lysophophatidylcholine
LPA	Lysophosphatidic acid
MCTs	Mono-carboxylate transporters
Meflin_CAF	Meflin-positive fibroblasts
MFA	Metabolite flux analysis
MS	Mass spectrometry
mt(ROS)	Mitochondrial reactive oxygen species
myCAF	Myofibroblasts
NEAAs	Non-essential amino acids
NFs	Normal fibroblasts
OCR	Oxygen consumption rate
OXPHOS	Oxidative phosphorylation
PDAC	Pancreatic ductal adenocarcinoma
PSCs	Pancreatic stellate cells
ROS	Reactive oxygen species
TCA	Tricarboxylic acid cycle
TME	Tumor microenvironment
UPLC	Ultra-performance liquid chromatography
